# Eutrophication and Deoxygenation Drive High Methane Emissions from a Brackish Coastal System

**DOI:** 10.1021/acs.est.4c00702

**Published:** 2024-06-05

**Authors:** Olga M. Żygadłowska, Florian Roth, Niels A. G. M. van Helmond, Wytze K. Lenstra, Jessica Venetz, Nicky Dotsios, Thomas Röckmann, Annelies J. Veraart, Christian Stranne, Christoph Humborg, Mike S. M. Jetten, Caroline P. Slomp

**Affiliations:** † Department of Earth Sciences—Faculty of Geosciences, Utrecht University, Princetonlaan 8a, 3584 CB Utrecht, The Netherlands; ‡ Baltic Sea Centre, Stockholm University, SE-106 91 Stockholm, Sweden; § Department of Microbiology, Radboud Institute for Biological and Environmental Sciences, Radboud University, 6525 AJ Nijmegen, The Netherlands; ∥ Institute for Marine and Atmospheric Research Utrecht, Utrecht University, 3584 CC Utrecht, The Netherlands; ⊥ Department of Aquatic Ecology and Environmental Biology, Radboud Institute for Biological and Environmental Sciences, Radboud University, 6525 AJ Nijmegen, The Netherlands; # Department of Geological Sciences, Stockholm University, SE-106 91 Stockholm, Sweden

**Keywords:** water column redox, sulfate–methane transition zone, organic carbon, sediment, sulfide

## Abstract

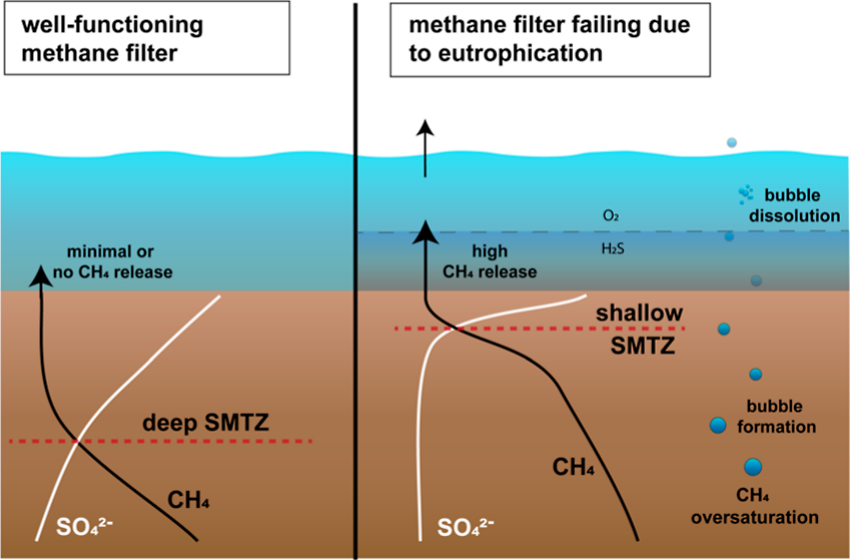

Coastal environments are a major source of marine methane in the atmosphere. Eutrophication and deoxygenation have the potential to amplify the coastal methane emissions. Here, we investigate methane dynamics in the eutrophic Stockholm Archipelago. We cover a range of sites with contrasting water column redox conditions and rates of organic matter degradation, with the latter reflected by the depth of the sulfate–methane transition zone (SMTZ) in the sediment. We find the highest benthic release of methane (2.2–8.6 mmol m^–2^ d^–1^) at sites where the SMTZ is located close to the sediment–water interface (2–10 cm). A large proportion of methane is removed in the water column via aerobic or anaerobic microbial pathways. At many locations, water column methane is highly depleted in ^13^C, pointing toward substantial bubble dissolution. Calculated and measured rates of methane release to the atmosphere range from 0.03 to 0.4 mmol m^–2^ d^–1^ and from 0.1 to 1.7 mmol m^–2^ d^–1^, respectively, with the highest fluxes at locations with a shallow SMTZ and anoxic and sulfidic bottom waters. Taken together, our results show that sites suffering most from both eutrophication and deoxygenation are hotspots of coastal marine methane emissions.

## Abstract 2

Coastal systems are increasingly a source of the potent greenhouse gas methane. This study reports high methane emissions from a eutrophic coastal system suffering from deoxygenation.

## Introduction

Methane is a potent greenhouse gas with a present-day atmospheric concentration that is 2.5 times higher than that before the industrial era.^1^ Coastal systems are a key but poorly quantified source of marine methane emissions.^2,3^ Most methane in coastal systems is produced in the final step of organic matter degradation in the anoxic part of the sediment.^4^ Typically, the methane is subsequently either removed through oxidation by consortia of anaerobic methanotrophic archaea and sulfate-reducing bacteria in the so-called sulfate methane transition zone^5^ (SMTZ) or, if oxygen is present, by aerobic methanotrophic bacteria.^4,6^

Coastal eutrophication and deoxygenation can disrupt the tight balance between methane production and oxidation in sediments.^7,8^ Intense phytoplankton blooms and the associated elevated flux of organic matter to the seafloor may drive bottom water oxygen depletion^9,10^ and enhance methane production in the sediment. Evidence suggests that, when rates of methane production increase, methane removal in the sediment cannot always keep up with the supply and an upward shift of the SMTZ may be observed.^11,12^ This can be even more pronounced in brackish sediments, where sulfate concentrations are lower than in marine sediments. The shoaling of the SMTZ can also be exacerbated when sediment accumulation rates are increased, since this leads to faster burial of organic matter, hence providing more substrate for methanogens deeper in the sediment.^12,13^ Increased sediment accumulation rates can also shorten the residence time of methanotrophs in the SMTZ, hindering the buildup of sufficient biomass to consume the generated methane.^12^ Excessive production of methane in the sediments can lead to oversaturation of methane in the porewaters and formation of methane bubbles,^14,15^ which can bypass the sediment filter and escape into the water column. While some bubbles will dissolve in the water column, there is a chance of bubbles escaping to the atmosphere especially in shallow areas^16^ (<50 m). Overall, a shallow SMTZ is expected to lead to a less-efficient benthic methane filter and increase the potential for the release of methane to the overlying water.^11,17^

When methane is released from the sediment in dissolved form, its transport in the water column is controlled by turbulent diffusion through density-driven convection or wind-induced mixing.^18,19^ In fully mixed, oxic waters, when turbulent diffusion is fast, methane can be transported upward rapidly. Especially in shallow coastal waters, transport of methane can be faster than its removal through aerobic methanotrophy, leading to substantial methane emissions to the atmosphere.^20,21^ When a water column is stratified, turbulent diffusion is slower and an oxycline may develop with methane accumulating in the anoxic deeper water.^20,22^ In such systems, most removal of dissolved methane has been found to take place near the oxycline, where methane and oxygen meet.^23−25^ Gas bubbles can be an additional source of methane, as they rise from the sediments and dissolve in the water column. Direct measurements that capture spatial and temporal changes in bubble release are difficult because of the stochastic nature of bubble emissions.^26,27^ As a consequence, the contribution of bubbles to coastal methane emissions is not well known.

Eutrophication and deoxygenation can have strong effects on the efficiency of a microbial methane filter in coastal waters. Temperature and/or salinity-induced stratification can separate bottom waters from surface waters. In eutrophic systems, this lack of mixing can lead to oxygen depletion in the bottom water. Changing redox conditions in the water column can influence the microbial filter in various ways, for example, by changing the microbial community structure and its efficiency in removing methane.^28^ At present, we still lack sufficient knowledge to predict how methane emissions from coastal systems respond to environmental changes.

In this study, we investigate the effects of eutrophication and deoxygenation on methane dynamics in the waters and surface sediments of the Stockholm Archipelago. We present porewater analyses, which we use to determine the depth of the SMTZ and estimate rates of the benthic release of methane. Methane emissions to the atmosphere are quantified from continuous in situ measurements of methane in surface waters and direct in situ flux measurements. Methane isotope data are used to assess the role of aerobic vs anaerobic methane removal and dissolution of methane bubbles, while bubble seeps were quantified by using an echo sounder. We find the highest rates of methane release to the atmosphere at sites with a shallow SMTZ and anoxic and sulfidic bottom waters.

## Materials and Methods

### Study Area

**Figure 1:**
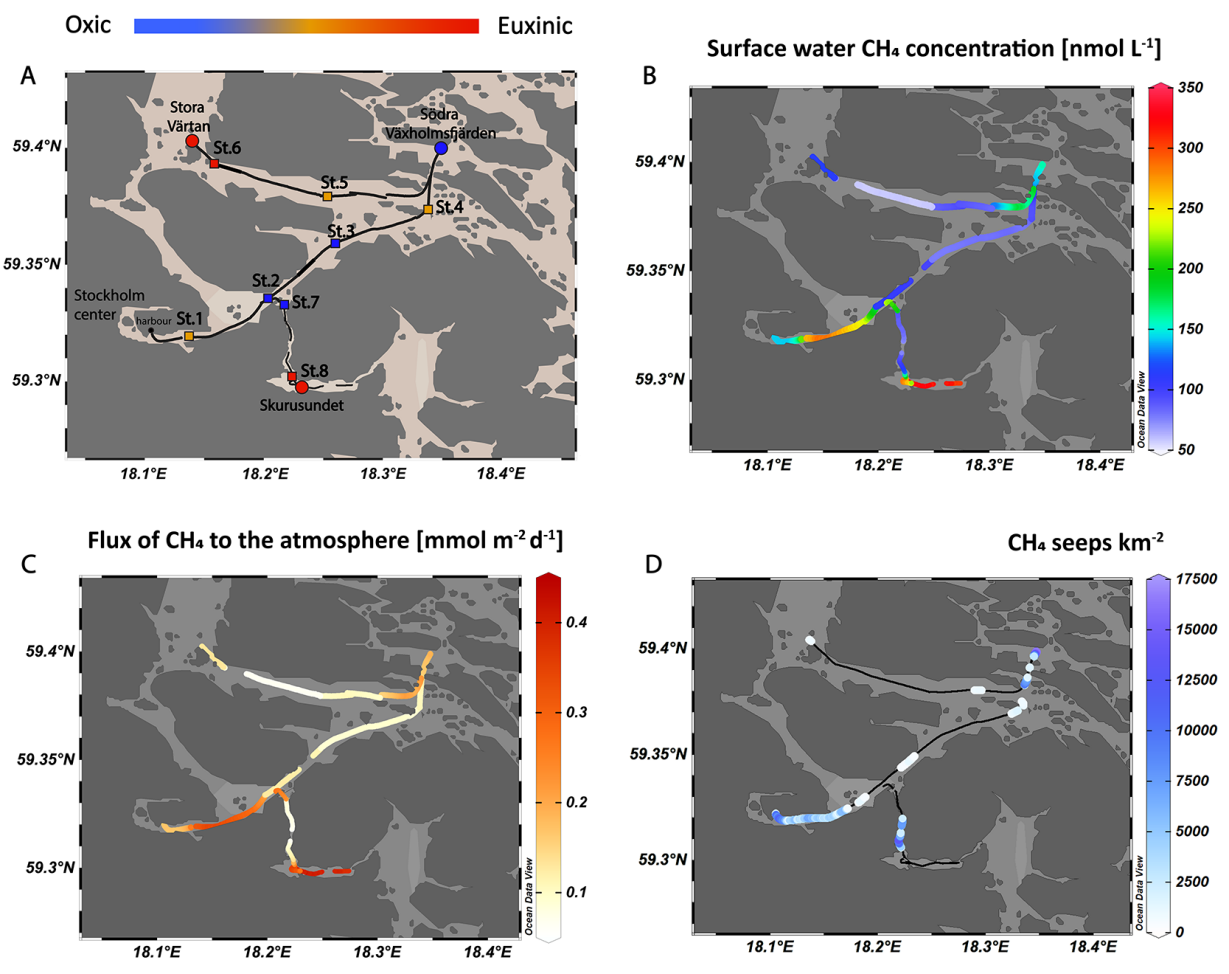
(A) Map of the study area in the Stockholm Archipelago. Continuous measurements with the water equilibration gas analyzer (WEGA) system and acoustic mapping were conducted along the transect indicated on the map as a black line. The main stations sampled in detail are marked with circles; additional transect stations are marked with squares. (B) Surface water–methane concentrations along the transect as measured continuously with the WEGA system while sailing. (C) Fluxes of methane to the atmosphere calculated based on the surface water–methane concentrations measured with the WEGA system. (D) Number of methane seeps per km^2^ obtained from the acoustic measurements. Seeps are visible as dots, whereas areas without seeps are indicated with a black line.

The Stockholm Archipelago is a eutrophic brackish system made up of a large number of primarily rocky islands, encircled by a network of basins and straits of various shapes, sizes, and depths.^29^ The sampling for this study was performed in the western part of the Stockholm Archipelago (Figure 1A). This region is characterized by a high input of fresh water from Lake Mälaren, a low surface water salinity (∼4.5), and mostly seasonally stratified waters.^30,31^ Wind action keeps the water column fully mixed only in more open parts of the archipelago.^32^ High inputs of nutrients have led to widespread eutrophication and associated water column hypoxia (oxygen <63 μmol L^–1^) and, in some areas, euxinia^33,34^ (oxygen = 0 μmol L^–1^, presence of sulfide). Many areas in the archipelago are characterized by high sedimentation rates and high sediment organic carbon contents, highlighting the eutrophic nature of this system.^35^

In this study, continuous measurements of surface water–methane concentrations and echo sounding for bubbles were performed along three transects in the Stockholm Archipelago in September 2022 with the RV Electra (Figure 1A). Sediment cores were taken at 11 sites (4 sites with oxic, 3 sites with hypoxic, and 4 sites with euxinic water column conditions; Table SA.1) to collect porewater for methane, sulfate, and sulfide analysis. Sediment organic carbon contents were determined on samples collected at the same sites in June 2019 (Section SA.1). High-resolution water column sampling for a range of chemical parameters, including methane isotopes and measurement of in situ sea–air fluxes, was performed at 3 of the 11 sites (henceforth termed the main sites). These sites are characterized by either seasonal (Södra Växholmsfjärden, Stora Värtan) or longer term euxinia (Skurusundet; Figure SA.1) and high organic carbon contents in the sediment (∼3–13 wt %; Figure SA.2). We note that at the time of sampling, the water column at Södra Växholmsfjärden was fully oxic, likely because of a recent renewal of the deeper water. Long-term monitoring data show that the water column at the site is seasonally euxinic and that there typically is a break in stratification in fall (Figure SA.1).

### Continuous Surface Water–Methane Concentration Measurements and Acoustic Mapping

The continuous measurements of methane concentrations in the surface waters were performed while sailing along three transects (Figure 1A) using a WEGA coupled to a cavity ring-down spectrometer (model G2131-I, Picarro Inc.). A detailed description of the WEGA system can be found in ref (35). The surface water–methane concentrations measured with WEGAs were used to calculate diffusive fluxes of methane to the atmosphere along the transects, as described in the Calculations section. Quantification of bubble seeps was conducted using a hull-mounted Simrad EK80 wideband split-beam scientific echo sounder with a center frequency of 70 kHz as installed on the RV Electra. A further description of the system and data processing can be found in ref (35).

### Sediment and Porewater Collection

Samples for methane analysis were collected from the first core immediately after core retrieval using a liner with predrilled holes at 2.5 cm intervals as described in Section SA.1.2. Note that methane degassing may occur during sample collection, leading to an underestimation of porewater–methane concentrations. While degassing intensifies with higher methane concentrations, changes in the isotopic composition of methane are not expected.^12,36^ A second core was sliced under a nitrogen atmosphere and processed further for the analysis of sulfate and sulfide in the porewater, as described in Section SA.1.3. At St.1, no sediment cores were taken. Instead, the methane and sulfate data published in ref (37) for the site “Strömmen” were used. A third sediment core was sliced to determine porosity based on the weight loss upon oven-drying, assuming a dry sediment density of 2.65 g cm^–3^.^38^

### Water Column Sampling

Depth profiles of dissolved oxygen, salinity, and temperature were obtained using a CTD Rosette (Seabird SBE 911 plus) equipped with 12 Niskin bottles that were used to collect water column samples (1–2 m resolution). For the analysis of methane and its isotopic composition (δ^13^C, δD), 120 mL serum bottles were filled from the bottom up directly from the Niskin bottle, while allowing them to overflow. The bottles were then quickly closed with rubber stoppers and crimped with aluminum caps, ensuring that no air bubbles remained inside. Directly after collection, the samples were poisoned with 0.25 mL saturated HgCl_2_ solution and stored upside down in the dark until the analysis. A description of the sample collection for sulfate, sulfide, and ammonium can be found in Section SA.1.4.

### Chemical Analyses

Prior to analysis for methane concentrations, 10 mL of nitrogen-gas headspace was added to all samples (water column and porewater) while the same amount of water. After gas- and water-phase equilibration (a minimum of 2 h for water column samples and 7 days for sediment samples), methane concentrations were measured with an HP 5890 series II gas chromatograph (Agilent Technologies with FID). The average analytical uncertainty based on replicate measurements was 2.4%. The isotopic composition of methane (δ^13^C, δD) for water column samples and porewater samples from the surface sediment was determined using continuous flow isotope ratio mass spectrometry as described previously.^39,40^ A description of the chemical analyses of sulfate, sulfide, and ammonium can be found in Section SA.1.5. The depth of the SMTZ in the sediments was determined as the depth of equimolar sulfate and methane concentrations.^41^

### In Situ Sea–Air Methane Flux Measurements

In situ measurements of the methane fluxes at the sea–air interface were performed at the three main stations (Södra Växholmsfjärden, Stora Värtan, and Skurusundet) with a cylindrical floating chamber (ø 390 cm, height 27 cm) connected to a LICOR trace gas analyzer (LI-7810). The measurements were performed in triplicate, with each measurement lasting 3–10 min to capture a linear increase in methane concentrations. A detailed description of the floating chamber method and its performance can be found in ref (28).

### Calculations

Diffusive fluxes of methane and sulfide across the sediment–water interface were calculated according to Fick’s first law
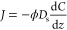
1where *J* is the diffusive flux in mmol m^2^ d^–1^, ϕ is the porosity of the sediment, *D*_s_ is the diffusion coefficient for methane and sulfide in the sediment in m^2^ d^–1^, *C* is the concentration of methane and sulfide in the porewater or bottom water in mmol m^–3^, and *z* is the sediment depth in m. *D*_s_ is calculated from the diffusion coefficient for methane and sulfide in seawater, corrected for salinity and temperature using the R package CRAN: *marelac*,^42^ accounting for the tortuosity of the sediment.^43^

Diffusive fluxes of methane across the water–atmosphere interface were calculated based on the following equation
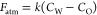
2where *F*_atm_ represents the diffusive flux from the water column to the atmosphere in mmol m^2^ d^–1^, *k* is the gas exchange coefficient in m d^–1^, *C*_W_ is the dissolved methane concentration at 1 m depth in mmol m^–3^, and *C*_O_ is the calculated methane concentration in equilibrium with the atmosphere in mmol m^–3^. Further details on the calculations are given in Section SA.2.

## Results and Discussion

### Variations in Bottom Water Oxygen and Sulfide in the Archipelago

The bottom water oxygen concentrations at the 11 sampling sites ranged from oxic (4 sites) and hypoxic (3 sites) to euxinic (4 sites; Table SA.1; Figure SA.3). Most of the oxic and hypoxic sites were located in a channel connecting central Stockholm to the rest of the archipelago, with hypoxia primarily occurring in more sheltered areas of the channel between small islands or close to a harbor (Figure 1A). Stratification and euxinia were encountered in shallow, enclosed areas with relatively little water exchange with the rest of the archipelago. High porewater sulfide concentrations near the sediment–water interface, corresponding high diffusive fluxes of sulfide to the overlying water, and the strong gradient in sulfate in the porewater (but not in the water column) highlight that the sediment is the major source of sulfide in the water column at the euxinic sites (Figure SA.2; Table SA.2). Long-term monitoring data (Figure SA.1) show that the water columns at Södra Växholmsfjärden and Stora Värtan are seasonally stratified with occasional bottom water euxinia developing over summer months, after which the water column mixes again in fall. At Skurusundet, however, a large portion of the water column remains stratified and sulfidic for extended periods of time, and full water column mixing occurs only every 2–4 years (Figure SA.1). This illustrates the negative impact of physical restriction on water quality in eutrophic coastal waters. The strong impact of eutrophication is further highlighted by the high ammonium concentrations (up to ∼200 μmol L^–1^) observed in the water column at all three main sites (Figure SA.4).

### Sea–Air Flux of Methane

The continuous surface water–methane measurements point toward particularly high methane concentrations and emissions to the atmosphere near the harbor in central Stockholm and in the Skurusundet area (>0.4 mmol m^–2^ d^–1^; Figure 1B,C). While the order of magnitude of the observed fluxes is comparable to that observed in other coastal systems in the Baltic Sea^44,45^ (0.1–0.2 mmol m^–2^ d^–1^), it is substantially higher than the average for continental shelves^46^ (0.03 mmol m^–2^ d^–1^). The key factors contributing to these high methane emissions are discussed in the following sections.

### Benthic Release of Methane

Methane can be released from the sediment in the form of bubbles and as dissolved methane. The results of the echo sounding revealed the highest density of methane bubble seeps (Figure 1D) in the area close to central Stockholm. Methane bubbles are formed in the sediment as a result of intense methane production and may be released to the overlying water either continuously or in discrete events.^35^ Upon escape to the water column, part of the methane bubbles will dissolve resulting in increased methane concentrations in surface waters. The release of methane bubbles from the sediment was also observed near Södra Växholmsfjärden and Stora Värtan but not at Skurusundet. Considering that waters in the archipelago are relatively shallow (<50m), it is likely that a fraction of the methane gas in the bubbles will be released directly to the atmosphere.^16^

**Figure 2:**
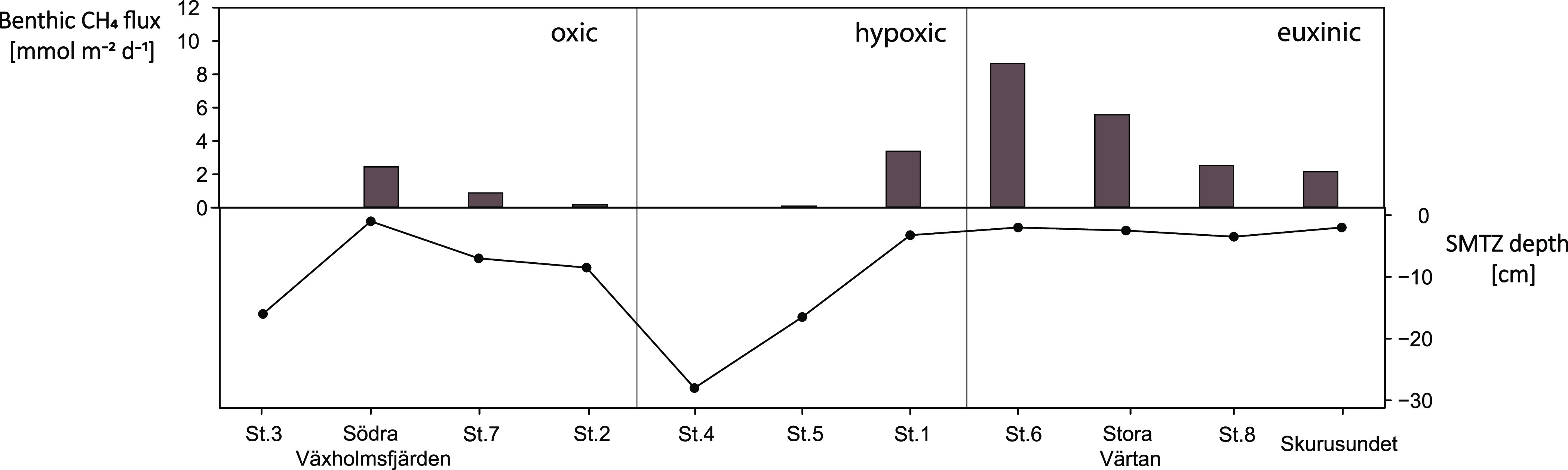
Methane fluxes at the sediment–water column interface and SMTZ depth for all of the study sites. The study sites are organized based on the bottom water oxygen and sulfide concentrations (from oxic to hypoxic to euxinic).

In the sediments of the eutrophic Stockholm Archipelago, the SMTZ is expected to be close to the sediment–water interface because of the low salinity and high input of organic matter.^13^ The porewater depth profiles of methane and sulfate (Figure SA.5) indeed reveal a shallow SMTZ (0–5 cm) at 6 of the 11 stations (Figure 2; Table SA.3). Anaerobic degradation of organic matter in sediments can lead to the release of reductants such as ammonium and sulfide to the water column, thereby contributing further to oxygen depletion in the bottom waters.^47^ We therefore expect the shallowest SMTZ to occur in areas with the highest organic matter input and anoxic bottom waters. In the Stockholm Archipelago, shallow SMTZ depths are mostly observed at stations with hypoxic or anoxic bottom waters. The only exception is Södra Växholmsfjärden. This station was oxic at the time of sampling but has a history of seasonal stratification and bottom water euxinia (Figure SA.1) and the water column was likely mixed shortly before our sampling.^48^

**Figure 3:**
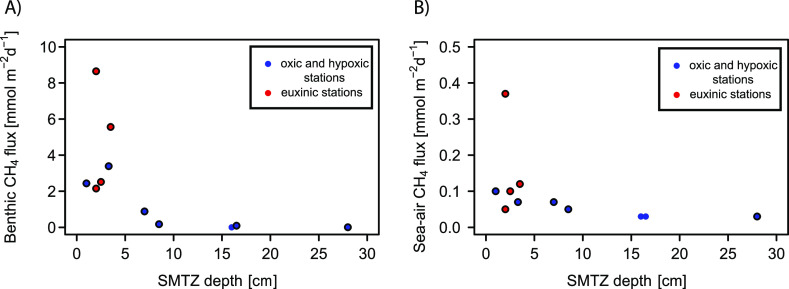
Relationship between the (A) benthic methane flux and the SMTZ depth and (B) sea–air methane flux and the SMTZ depth for oxic, hypoxic, and euxinic stations.

Benthic fluxes of methane varied strongly between the stations and ranged from ∼0 to 8.4 mmol m^–2^ d^–1^ (Figure 2). While high rates of benthic methane release at sites with a shallow SMTZ have been reported previously (e.g., marine Lake Grevelingen^12^ and brackish Pojo Bay^44^), our results nicely illustrate that benthic methane fluxes and SMTZ depth are inversely correlated (Figure 3A). This indicates that the benthic release of methane increases as the depth of SMTZ becomes more shallow. In the Stockholm Archipelago, SMTZ depths are very shallow (<5 cm) at many stations, which is likely due to a combination of many decades of eutrophication^34^ and the low salinity of the bottom water. As a consequence, abundant methane accumulates close to the sediment surface, leaving only a narrow layer in which sulfate is available as the electron acceptor for anaerobic methanotrophic consortia, which reduces the efficiency of the microbial filter^7,12^ (Figure 2). Such a shallow SMTZ can also lead to the release of methane to the water column when bottom waters are oxic, as illustrated by the results for Södra Växholmsfjärden (Figure 2).

### Methane Dynamics in Well-Mixed Oxic Waters

**Figure 4:**
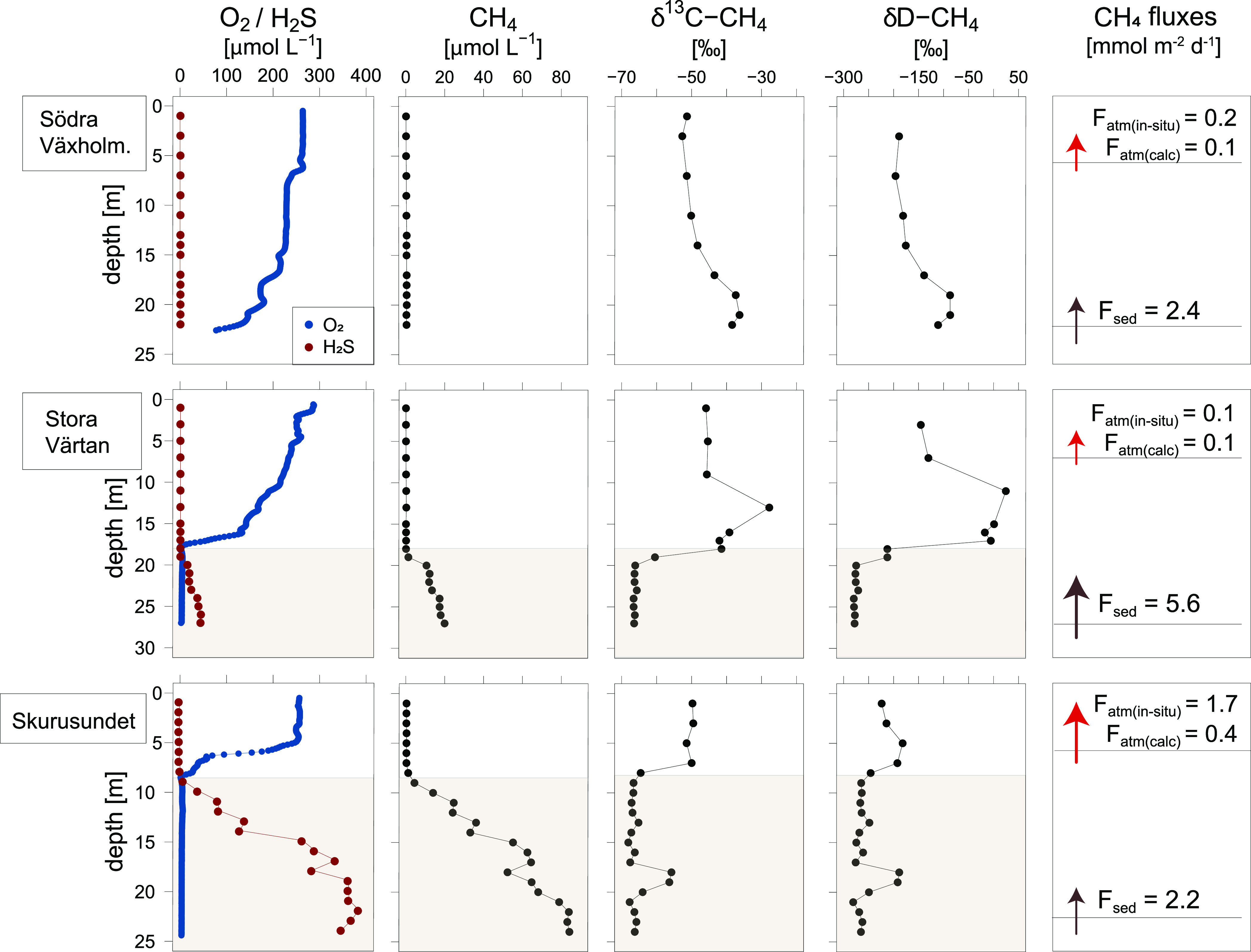
Depth profiles of oxygen, sulfide, methane, and methane isotopic composition (δ^13^C–CH_4_ and δD–CH_4_) in the water column of the three main stations. Shaded areas represent the euxinic parts of the water column. The last panel shows the in situ (*F*_atm(in situ)_) and calculated (*F*_atm(calc)_) flux of methane at the sea–air interface and the calculated diffusive flux at the sediment–water interface (*F*_sed_).

Methane concentrations in the oxic and sulfide-free water column of Södra Växholmsfjärden ranged from 0.1 to 0.5 μmol L^–1^ with the highest concentrations observed in the bottom waters (Figure 4). An enrichment in δ^13^C–CH_4_ and δD–CH_4_ relative to the source signature in the porewaters (−58.7 and −262‰, respectively; Table SA.4) was observed in the bottom waters suggesting methane oxidation close to the sediment–water interface. Despite the abundant presence of oxygen in the water column at this site, methane was released to the atmosphere at an in situ-measured rate of 0.2 mmol m^–2^ d^–1^. When assuming diffusive supply from the sediments only (estimated at 2.4 mmol m^–2^ d^–1^; Figure 4) and no other sources of methane, this would imply oxidative removal of the majority (estimated at 92%) of the methane released from the sediment in the water column, and escape of the remainder (i.e., 8%) to the atmosphere.

However, as noted above, water column methane was also supplied from bubble dissolution at this site (Figure 1D). Bubbles that rise up from the sediment carry the ^13^C-depleted isotopic signature of the methane in the sediment porewater, and their dissolution in the water column leads to a shift in the isotopic composition of the dissolved methane toward more negative values. At Södra Växholmsfjärden, the gradual decrease in δ^13^C–CH_4_ toward the surface, with a final surface water signature of −52%, suggests that over half (68%) of the methane in the surface water is supplied due to bubble dissolution (Table 1; Section SA.4). A lower abundance of methanotrophs is often observed in surface waters of coastal systems when compared to the deeper waters.^25^ Hence, with less methanotrophic activity in the surface waters, bubble dissolution close to the sea–air interface may enhance the release of methane to the atmosphere. The decreased efficiency of the microbial filter in the water column at Södra Växholmsfjärden is likely a result of both enhanced transport of methane toward the surface waters by turbulent diffusion and an increased methane supply from bubble dissolution.

**Table 1:** Estimation of the Contribution of Bubble Dissolution to Methane Concentrations at 5 m Depth Based on the Methane Isotope Data and Mass Balance (Supporting Information, Section SA.4)

station	methane concentration at 5 m	contribution from diffusion	contribution from bubble dissolution	
	(μmol L^–1^)	(μmol L^–1^)	(μmol L^–1^)	(%)
Södra Växholmsfjärden	0.15	0.05	0.10	67
Stora Värtan	0.10	0.05	0.05	45
Skurusundet	0.39	0.36	0.03	9

### Methane Dynamics in Stratified Waters with Euxinic Bottom Waters

The water column at the two other main stations, namely, Stora Värtan and Skurusundet, was stratified and characterized by euxinic bottom waters at the time of sampling. At Stora Värtan, sulfide and methane concentrations increased to values of up to 43 and 20 μmol L^–1^, respectively, below the oxycline, which was located at a depth of 17 m (Figure 4). The δ^13^C–CH_4_ value at and above the oxycline was more positive, with the highest value observed at a depth of 13 m (−28‰). Taken together, these results suggest that most methane was removed aerobically above the oxycline. The fractionation factor calculated for the zone where enrichment in δ^13^C–CH_4_ was observed (1.0107; Section SA.3; Table SA.5) was within the range of fractionation factors reported for aerobic and anaerobic methane oxidation (1.002–1.035; Grant and Whiticar 2002). Aerobic removal near the oxycline is typically the major pathway of methane oxidation in stratified coastal systems.^25,49,50^ The presence of an oxycline is generally considered to have a positive effect on the efficiency of microbial methane removal.^20^ When assuming a benthic release of dissolved methane of 5.6 mmol m^–2^ d^–1^ and a sea–air flux of methane of 0.1 mmol m^–2^ d^–1^, the efficiency of methane removal at Stora Värtan would be high (estimated at 98%). Similar to Södra Växholmsfjärden, however, there was a substantial supply of methane to the surface waters from bubble dissolution (45% of measured methane concentrations at 5 m depth; Table 1), which likely contributed to methane emissions to the atmosphere. Still, the strong density stratification will have increased the efficiency of methane removal by slowing down turbulent transport across the oxycline, giving the methanotrophs ample time to remove the incoming methane. While methane emissions were low at the time of sampling, the benefit of stratification was likely only temporal, as in seasonally stratified basin methane that accumulates below the oxycline can be emitted to the atmosphere upon water column mixing.^20^

At Skurusundet, oxygen was already depleted below a water depth of 8 m, and sulfide was present throughout the anoxic part of the water column with concentrations reaching values of nearly 400 μmol L^–1^ near the seafloor (Figure 4). Similarly, methane concentrations increased with water depth, reaching values up to 80 μmol L^–1^ in the bottom waters. The depth profiles of δ^13^C–CH_4_ and δD–CH_4_ show the most positive δ^13^C and δD values in the oxic and anoxic parts of the water column (upper 7 m and at ∼19 m). Although the ^13^C enrichment observed in the anoxic waters might be indicative of anaerobic methane removal, its quantitative contribution to total methane removal is likely limited in sulfide-rich deeper water. Lateral input of water with methane with a different isotopic signature could also explain such a ^13^C-enrichment. The positive δ^13^C and δD values directly above the oxycline are likely the result of aerobic methane oxidation, even though the shift in the isotopic signatures was less pronounced than that at the other stations (maximum of −49.5‰ for δ^13^C–CH_4_; Figure SA.5). Here, similar to Stora Värtan, the fractionation factor for δ^13^C – CH_4_ also points to aerobic methane oxidation (1.0065; Table SA.5). Importantly, no bubble seeps were observed at this station (Figure 1D), suggesting a little bubble release. Taken together, this implies that the methanotrophic activity here was weaker than at the other stations. We hypothesize that the efficiency of aerobic methane removal was limited because of strong wind-induced mixing of the surface waters. This mixing would not only enhance the upward transport of methane but also hinder the establishment of a stable methanotrophic community.^28^ Apparently, such a narrow and turbulent oxic layer of the water column does not provide suitable conditions for efficient aerobic methane oxidation. Assuming that the benthic flux of 2.2 mmol m^–2^ d^–1^ captures the total release of methane from the sediment, the efficiency of methane removal at Skurusundet is only 23% with 1.7 mmol m^–2^ d^–1^ of methane (77%) escaping to the atmosphere. As already noted, the contribution of methane from bubble dissolution in the surface waters in this area was low (Figure 1D). Based on the isotope balance, we estimate a contribution of 9% (Table 1; Section SA.4). In addition to the presence of shallow oxyclines, high sulfide concentrations in the water column also may affect methane removal. High sulfide concentrations have been observed to hinder the activity of anaerobic methanotrophs in sediments.^51^ Given the toxicity of sulfide, impacts on aerobic methanotrophic communities in the water column could also be expected. We conclude that the longer-term stratification and the development of shallow water column euxinia likely play key roles in limiting methane removal at Skurusundet, thereby promoting high methane release to the atmosphere.

### Highest Methane Emissions from Sites with a Shallow SMTZ and Euxinic Waters

To assess whether the depth of the SMTZ also impacts atmospheric fluxes of methane, we plotted the calculated sea–air fluxes of methane as a function of the SMTZ depth for the 11 stations along the transects. Similar to the trend for the benthic fluxes (Figure 3A), we observe an inverse correlation between the depth of the SMTZ and methane emissions to the atmosphere (Figure 3B), i.e., the shallower the SMTZ, the higher the flux of methane from the surface water to the atmosphere. This anticorrelation is particularly evident for the oxic stations, indicating that at sites with a fully mixed water column, a failure of the microbial filter in the sediment directly contributes to higher methane emissions to the atmosphere. Stations with a stratified water column were generally characterized by a shallow SMTZ, but we find both high and low fluxes of methane to the atmosphere at such stratified stations (Figure 3B). In a stratified water column, the density differences lead to formation of physical barriers that slow down the vertical transport of methane, thereby providing more time for methanotrophs to remove methane.^24^ In enclosed areas that remain stratified for extended periods of time, the efficiency of the microbial filter appears to be compromised by the limited supply of oxygen and the high sulfide concentrations at depth and strong mixing in the oxic surface layer. Hence, while the efficiency of the microbial filter can be decreased under both oxic and anoxic/euxinic conditions, the mechanisms behind this decrease in efficiency are different. This highlights the role of the interaction between the physical and microbial processes in controlling methane removal in coastal systems.

Overall, we find that at sites with a shallow SMTZ, the microbial methane filter in the sediment has become less efficient, leading to a higher level of benthic methane release. In the water column, in turn, the efficiency of the microbial filter is strongly affected by stratification and the prevailing redox conditions. While the release of methane to the atmosphere was observed at sites with either a well-mixed or stratified water column, the weakest microbial filter and hence the highest methane emissions were observed in an area with longer-term stratification, a shallow oxycline, and high sulfide concentrations in the water column. We conclude that eutrophication and deoxygenation have the potential to greatly enhance the emissions of methane from coastal waters to the atmosphere. Thus, water management of coastal ecosystems should be directed toward reductions in nutrient inputs and restoration of oxygen levels to prevent further methane emissions.

## Data Availability

The original contributions presented in the study are included in the article/Supporting Information and in the Zenodo repository (https://doi.org/10.5281/zenodo.10959090), further inquiries can be directed to the corresponding author.

## Supporting Information Available

Sediment collection and analysis for total organic carbon; detailed descriptions of the porewater–methane, sulfate and sulfide sampling; detailed description of the water column sulfate, sulfide and ammonium sampling and analysis; detailed descriptions of the diffusive water–atmosphere methane flux calculations; fractionation factor calculations. The contribution of methane from bubble dissolution calculations. Figures: long-term monitoring data for water column oxygen and sulfide for the three main stations; porewater sulfide and sediment organic carbon with depth in the sediments, oxygen, temperature and salinity depth profiles for all sites; water column depth profiles of ammonium and sulfate; porewater–methane and sulfate for all sites; and isotopic composition of methane at the three main sites. Tables: General characteristics of the study sites; diffusive fluxes of sulfide at the sediment–water interface; SMTZ depths and the fluxes of methane to the atmosphere for all sites; methane isotopes in the porewaters of the top sediments at the three main stations; and fractionation factors for the water column methane isotopes (PDF)
