# Supplementary material for: Eutrophication and Deoxygenation Drive High Methane Emissions from a Brackish Coastal System
Source: Environ Sci Technol. 2024 Jun 5;58(24):10582–90. doi: 10.1021/acs.est.4c00702 (PMC11191596; doi:10.1021/acs.est.4c00702)
Supplement: Supplementary file 1 — es4c00702_si_001.pdf [file es4c00702_si_001.pdf]

Supporting Information

Eutrophication and deoxygenation drive high methane emissions from a brackish coastal system

Olga M. Żygadłowska[1], Florian Roth[2], Niels A.G.M. van Helmond[1,3], Wytze K. Lenstra[1,3], Jessica Venetz[3], Nicky Dotsios[3], Thomas Röckmann[4], Annelies J. Veraart[5], Christian Stranne[2,6], Christoph Humborg[2], Mike S.M. Jetten[3], Caroline P. Slomp[1,3]

[1] Department of Earth Sciences - Faculty of Geosciences, Utrecht University, Princetonlaan 8a, 3584 CB Utrecht, The Netherlands

[2] Baltic Sea Centre, Stockholm University, SE-106 91 Stockholm, Sweden

[3] Department of Microbiology, Radboud Institute for Biological and Environmental Sciences, Radboud University, 6525 AJ Nijmegen, The Netherlands

[4] Institute for Marine and Atmospheric Research Utrecht, Utrecht University, 3584 CC Utrecht, The Netherlands

[5] Department of Aquatic Ecology and Environmental Biology, Radboud Institute for Biological and Environmental Sciences, Radboud University, 6525 AJ Nijmegen, The Netherlands

[6] Department of Geological Sciences, Stockholm University, SE-106 91 Stockholm, Sweden

Number of pages: 14

Number of tables: 5

Number of figures: 6

Number of equations: 6

Table SA.1. General characteristics of the study sites.

Station	Coordinates	BW redox conditions	Water depth [m]	BW O_2 / H_2S [µmol L^{-1}]	BW Salinity	BW Temperature [°C]
**St.3**	N 59°21.645'; E 18°15.538'	oxic	44	84/0	5.4	6
**Södra Växholmsfjärden**	N 59°23.934'; E 18°20.796'	oxic	24.5	81/0	4.6	13.5
**St.7**	N 59°19.911'; E 18°13.047'	oxic	36	66/0	5.3	7
**St.2**	N 59°20.018'; E 18°11.866'	oxic	43	63/0	5.4	6.1
**St.4**	N 59°22.445'; E 18°20.284'	hypoxic	32.5	53/0	5.3	8.7
**St.5**	N 59°22.763'; E 18°14.896'	hypoxic	30	34/0	5.2	7.8
**St.1**	N 59°19.125'; E 18°07.837'	hypoxic	33	31/0	5.3	7.1
**St.6**	N 59°23.563'; E 18°09.633'	euxinic	28.5	<2 / n.a.*	5.2	5.6
**St.8**	N 59°18.102'; E 18°13.502'	euxinic	16	<2 / n.a.*	4.3	5
**Stora Värtan**	N 59°24.252'; E 18°08.198'	euxinic	29	<2 / 43	5.1	4.8
**Skurusundet**	N 59°17.902'; E 18°13.764'	euxinic	26.5	<2 / 349	4	4.9

BW: bottom water

* smell of H_2S detected

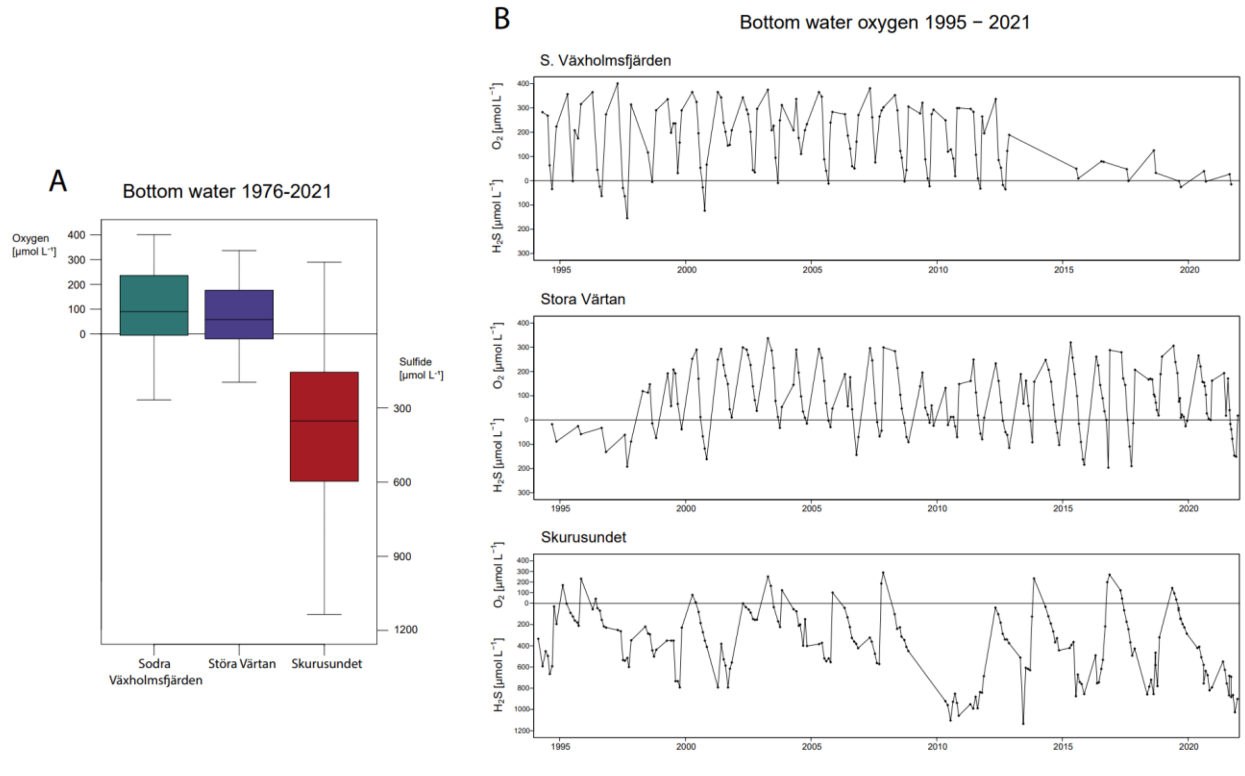

Figure SA.1. Long term monitoring data for oxygen and sulfide (obtained from the Swedish Meteorological and Hydrological Institute; SharkWeb (smhi.se)) for Södra Växholmsfjärden, Stora Värtan and Skurusundet. A) Mean values and range for 1976 to 2021. B) Seasonal cycle for 1995 – 2021. Note that oxygen and sulfide concentrations are plotted in opposite directions.

Section SA.1.1. Sediment collection and analysis for total organic carbon.

During a sampling trip in July 2019 on board R/V Electra a sediment core was collected with a Gemini gravity corer at Södra Växholmsfjärden, Stora Värtan and Skurusundet and sliced under an N_2 atmosphere, at a resolution of 0.5 cm (0-2 cm), 1 cm (2-10 cm), 2 cm (10-20 cm), 4 cm (20-40 cm) and 5 cm until the bottom of the core. Each slice was placed in a centrifuge tube and stored in N_2-flushed gas-tight aluminum bags at -20°C until further analysis. The sediment samples were subsequently freeze-dried, powdered and homogenized using an agate mortar and pestle. Between 200 and 300 mg of the oxic sediment split was decalcified using 1M HCl, as described by van Helmond et al. (2018), after which dried and repowdered residues were analyzed for their carbon content with a Fisons Instruments NA 1500 NCS analyzer. Results were normalized to inhouse standards. The average uncertainty of duplicate samples was 0.08 wt.%. Total organic carbon (TOC) contents were calculated after a correction for weight loss during decalcification (Van Santvoort et al., 2002).

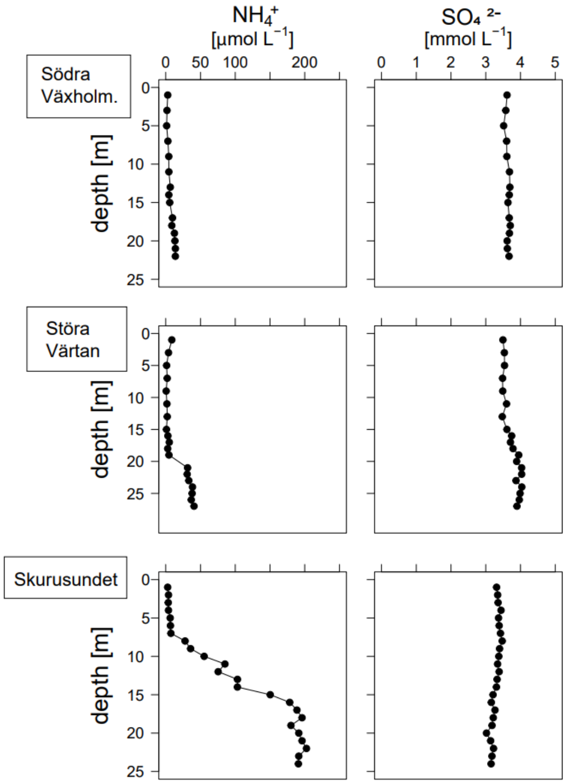

Figure SA.2. Depth profiles of sulfide and TOC in the sediments at the three main stations. The TOC data were obtained in July 2019.

Section SA.1.2. Detailed description of the porewater methane sampling

At each site, three sediment cores were collected using a UWITEC gravity corer and transparent PVC core liners (120 cm length, 6 cm inner diameter). Samples for methane analysis were collected from the first core immediately after core retrieval using a liner with pre-drilled holes at 2.5 cm intervals and covered with tape. Per depth, 10 mL of sediment was extracted with a modified plastic syringe and transferred into a 65 mL glass bottle filled with saturated NaCl solution. The bottle was topped up with NaCl, sealed, and stored upside down until analysis.

Section SA.1.3. Detailed description of the porewater collection for the sulfate and sulfide analysis

For the main sites, the second core was sliced (at 1 cm resolution) in a glovebag under a nitrogen atmosphere. Each slice was placed in a centrifuge tube. Selected samples (each sample for the 0-

10 cm interval, every second sample for the 10-30 cm interval and every fifth sample for the 40-90 cm interval) were centrifuged at 4500 rpm for 20 minutes to extract the porewater. The porewater was then filtered over 0.45 µm nylon filters and subsampled for a range of solutes. The subsamples for sulfate analysis were stored at 4°C. The subsamples for sulfide analysis were stored in a glass vial with a 2% zinc acetate solution at 4°C. For the transect sites, samples were collected with syringes as used for the methane analysis, albeit, here the samples were directly transferred into centrifuge tubes. All the samples were centrifuged, filtered over 0.45 µm nylon filters and stored at 4°C.

Section SA.1.4. Detailed description of the water column sampling for the sulfate, sulfide and ammonium

At the three main stations samples for sulfate, sulfide and ammonium were filtered through 0.2 µm nylon syringe filters. Samples for sulfate were acidified with 35% HCl (10 µL per 1 mL of sample) and stored at 4°C. Samples for sulfide were stored in a glass vial with a 2% zinc acetate solution at 4°C. Samples for ammonium were stored at -20°C.

Section SA.1.5. Chemical analyses of sulfate, sulfide and ammonium

Sulfate concentrations in the porewater were measured with ion chromatography (detection limit <50 µmol L^{-1}, average analytical uncertainty based on duplicates <5%). Sulfate concentrations in the water column were estimated from total dissolved sulfur concentrations determined using Inductively Coupled Plasma Optimal Emission Spectroscopy (ICP-OES, Perkin-Elmer Avio 500, detection limit 9.6 µmol L^{-1}). Sulfide concentrations were determined using the phenylenediamine and ferric chloride method[41]. Ammonium concentrations were determined using the indophenol blue method (Solorzano, 1969).

Section SA.2. Detailed description of the diffusive fluxes of methane across the water-atmosphere interface calculations.

The dissolved concentrations of methane in seawater (C_W) and air (C_O) were calculated as:

$$C_W = xCH_{4sw}\, \beta\, \rho \qquad \text{(Equation SA.1)}$$

$$C_O = xCH_{4atm}\, \beta\, \rho \qquad \text{(Equation SA.2)}$$

where xCH_{4sw} and xCH_{4atm} are the measured molar fractions of methane in sea water and atmosphere in nmol mol^{-1}, β represents the Bunsen solubility coefficient (dimensionless) and ρ is

the atmospheric pressure in bar. The Bunsen solubility coefficient is calculated from Wiesenburg and Guinasso (1979) as:

$$ln\beta = A1 + A2\left(\frac{100}{T}\right) + A3\ln\left(\frac{T}{100}\right) + S[(B1 + B2\left(\frac{T}{100}\right) + B3\left(\frac{T}{100}\right)^2] \quad \text{(Equation SA.3)}$$

where A1, A2, A3, B1, B2 and B3 are constants, T is the temperature in K and S is salinity. The gas exchange coefficient (k) is determined according to Wanninkhof (2014):

$$k = 0.251\, U^2 \left(\frac{Sc}{600}\right)^{-0.5} \quad \text{(Equation SA.4)}$$

where U is the wind speed in m s^{-1}, Sc is the Schmidt number (dimensionless), which is the quotient of kinematic viscosity of seawater in m^2 s^{-1} which is dependent on temperature and salinity (Pilson, 2013) and the diffusion coefficient of methane in seawater in m^2 s^{-1}, dependent on temperature (Jähne et al., 1987). The minimum and maximum wind speed data for each sampling day were obtained from the Swedish Meteorological and Hydrological Institute (SMHI) monitoring database for the nearest meteorological station (station Stockholm – Observatoriekullen; https://www.smhi.se/data/meteorologi/vind). The calculated minimum and maximum flux of methane to the atmosphere was then averaged for each site.

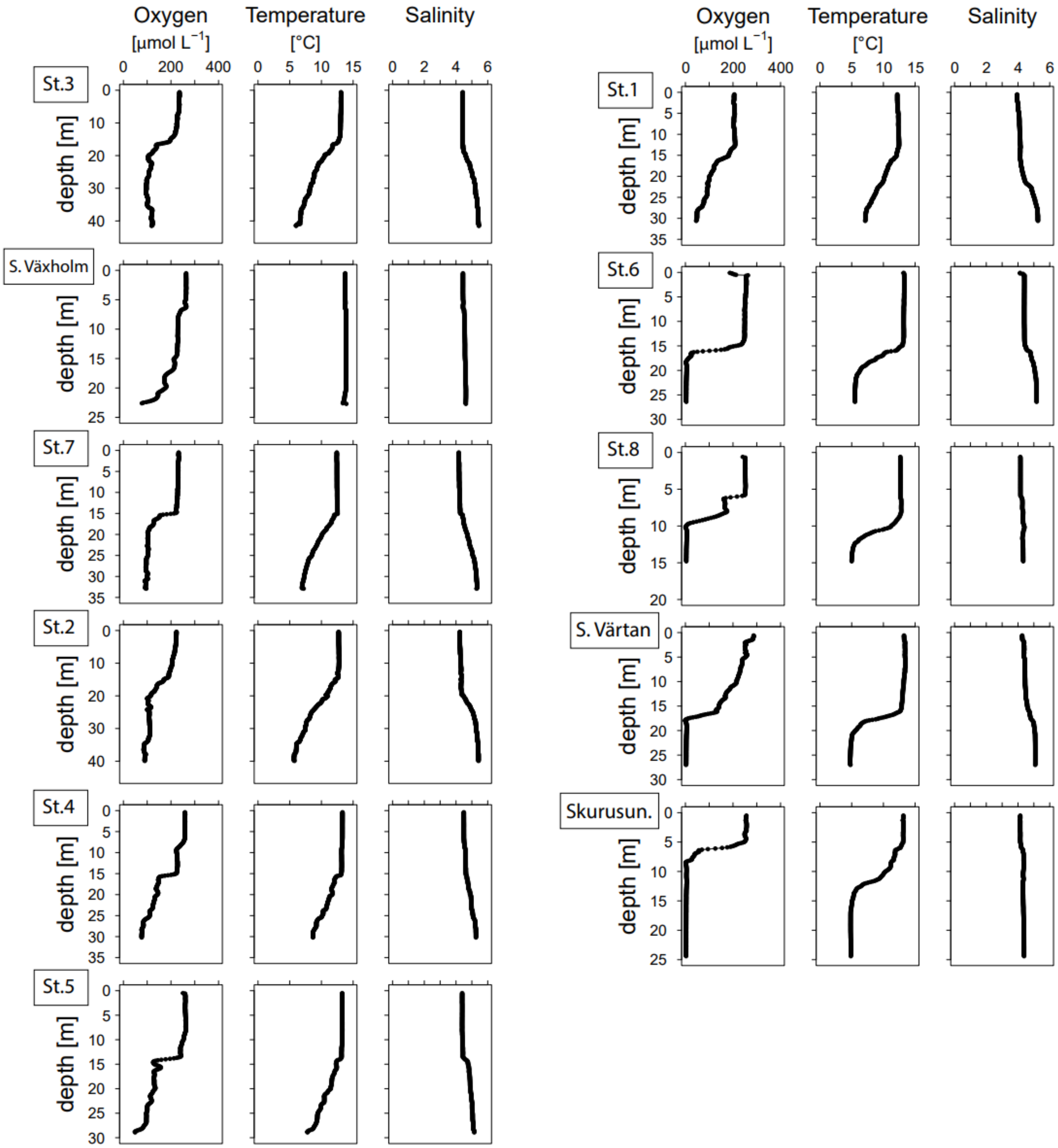

Figure SA.3. Depth profiles of oxygen, temperature and salinity for all the sites.

Table SA.2. Diffusive fluxes of sulfide at the sediment-water interface at the three main sites.

	H_2S flux [mmol m^{-2} d^{-1}]
**Södra Växholmsfjärden**	14.7
**Stora Värtan**	11.8
**Skurusundet**	13.2

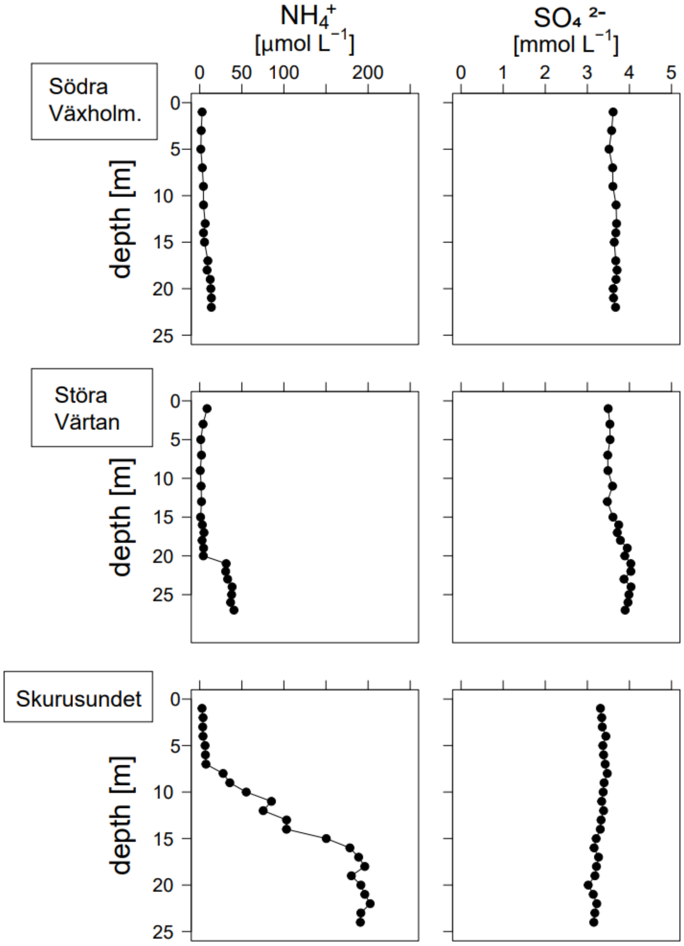

Figure SA.4. Water column profiles of ammonium and sulfate at the three main sites illustrating the eutrophic nature of the water column and the lack of major changes in sulfate concentrations with water depth.

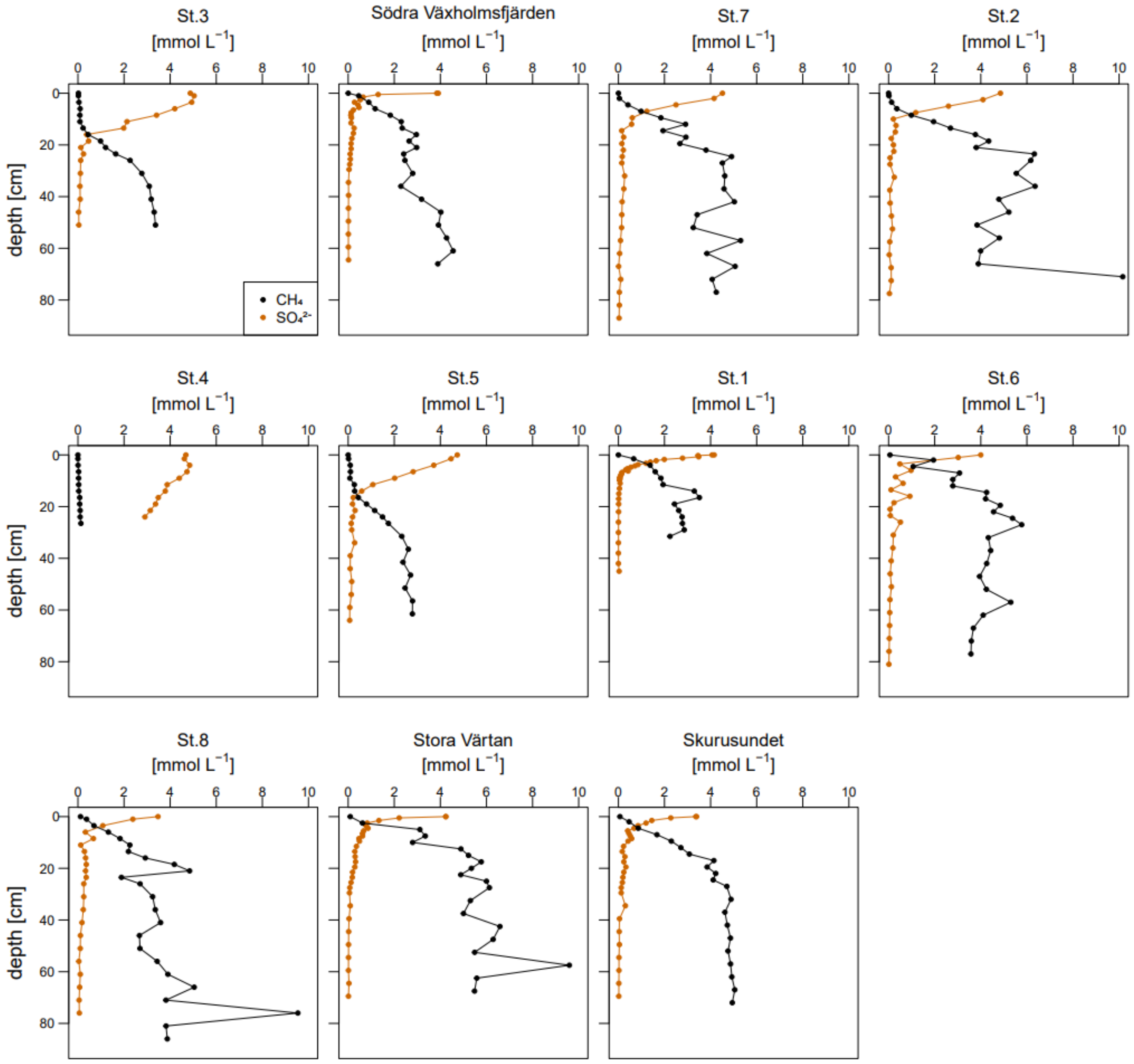

Figure SA.5. Porewater methane and sulfate concentrations at all sites in the Stockholm Archipelago.

Table SA.3. The SMTZ depth and the in-situ and calculated fluxes of methane to the atmosphere for all the stations.

Station	SMTZ depth [cm]	Flux of methane to the atmosphere [mmol m^{-2} d^{-1}]	
		**In-situ**	**Average calculated**
St.3	16.0		0.03
Södra Växholmsfjärden	1.0	0.19	0.10
St.7	7.0		0.07
St.2	8.5		0.05
St.4	n.d.		0.03
St.5	16.5		0.03
St.1	3.3		0.07
St.6	2.0		0.05
St.8	3.5		0.12
Stora Värtan	2.5	0.13	0.10
Skurusundet	2.0	1.68	0.37

n.d.: not detected - At St. 4 the SMTZ was located below the last sampled depth.

Table SA.4. Methane isotopes in the porewater in the upper 5 cm of the sediments at the three main stations.

	$\delta^{13}C$ – CH_4 [‰]	δD – CH_4 [‰]
**Södra Växholmsfjärden**		
bottom water	-58.7	-262
1 cm	-63.7	-279
3.5 cm	-63.3	-279
**Stora Värtan**		
bottom water	-60.4	-293
2.5 cm	-58.8	-295
5 cm	-56.6	-292
**Skurusundet**		
bottom water	-66.4	-283
2 cm	-66.0	-292
4.5 cm	-65.5	-291

Section SA.3. Fractionation factors

The fractionation factors were derived from the equation as described in Grant and Whiticar (2002):

$$\delta^{13}C - CH_4 = \left(\frac{1}{\alpha} - 1\right) \ln f + (\delta^{13}C - CH_4)_0 \qquad \text{(Equation SA.5)}$$

where $\delta^{13}C - CH_4$ is the isotopic signature of the methane, α is the fractionation factor, f is the fraction of methane remaining and $(\delta^{13}C - CH_4)_0$ is the isotopic composition of methane before oxidation. The parameter f was calculated based on the concentrations of methane at depths of 13 and 20 m for Stora Värtan and 7 and 9 m for Skurusundet.

Table SA.5. Fractionation factors for the water column methane isotopes

	**Fractionation factor**
Stora Värtan	1.0107
Skurusundet	1.0065

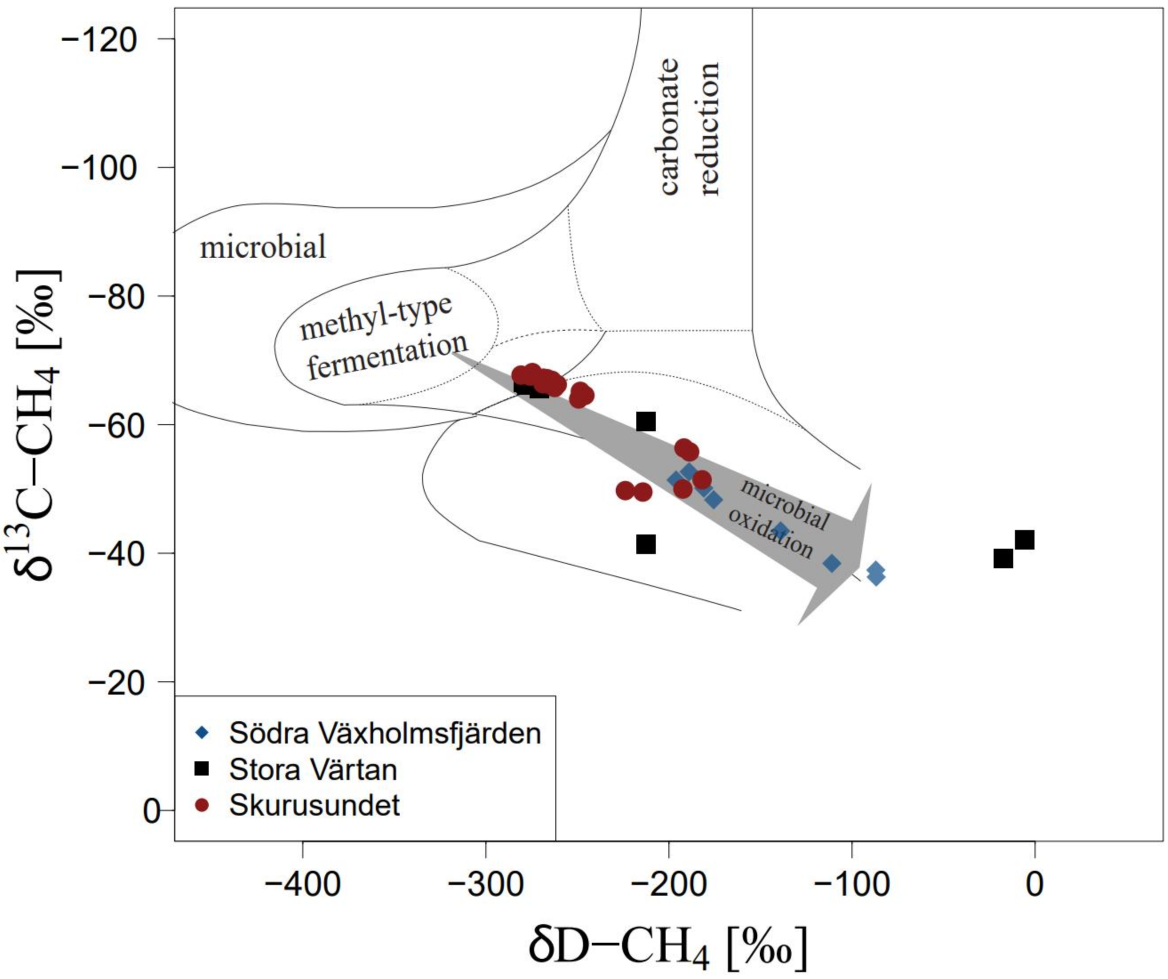

Figure SA.6. Isotopic composition of methane (δ13C-CH4 and δD-CH4) at the three main stations, with a background modified after (Whiticar, 1999; Egger et al., 2017). The grey arrow indicates the direction of the shift in isotopic composition due to microbial methane oxidation (Whiticar, 1999).

Section SA.4. The contribution of methane bubble dissolution to dissolved methane concentrations in the surface waters

To calculate the contribution of bubble dissolution to total methane concentrations at 5 m we applied a simple mass balance:

$$C_s Iso_s = C_{diff} Iso_{diff} + C_{bubb} Iso_{bubb} \quad \text{(Equation SA.6)}$$

where C_s, Iso_s, C_{diff}, Iso_{diff}, C_{bubb}, Iso_{bubb} are the concentrations and isotopic signatures of methane in the surface waters, supplied from diffusion and supplied from bubble dissolution, respectively. To calculate the unknown terms in the mass balance (C_{diff} and C_{bubb}) we assumed that the sample with the strongest isotopic enrichment represents the signature of methane supplied from diffusion and that the sample for the depth closest to the sediment-water interface represents the signature of methane supplied from bubble dissolution.

REFERENCES

Egger, M., Hagens, M., Sapart, C. J., Dijkstra, N., van Helmond, N. A. G. M., Mogollón, J. M., et al. (2017). Iron oxide reduction in methane-rich deep Baltic Sea sediments. Geochim. Cosmochim. Acta 207, 256–276. doi: 10.1016/j.gca.2017.03.019.

Grant, N. J., and M. J. Whiticar. 2002. Stable carbon isotopic evidence for methane oxidation in plumes above Hydrate Ridge, Cascadia Oregon Margin. Global Biogeochem. Cycles 16: 71-1-71–13. doi:10.1029/2001gb001851

Jähne, B.; Heinz, G.; Dietrich, W. (1987). Measurement of the Diffusion Coefficients of Sparingly Soluble Gases in Water. J. Geophys. Res. Ocean. 92 (C10), 10767–10776. doi: 10.1029/JC092iC10p10767.

Pilson, M. E. Q. (2013). An Introduction to the Chemistry of the Sea; Cambridge University Press. doi: 10.1017/CBO97381139047203.

Solorzano L. (1969). Determination of ammonia in natural waters by the phenolhypochlorite method. Limnol. Oceanogr. 14 (5), 799-801. doi: 10.4319/lo.1969.14.5.0799.

Van Helmond N. A. G. M., Jilbert, T., and Slomp, C. P. (2018). Hypoxia in the Holocene Baltic Sea: Comparing modern versus past intervals using sedimentary trace metals. Chem. Geol. 493, 478–490, doi: 10.1016/j.chemgeo.2018.06.028, 2018.

Van Santvoort P. J. M., De Lange G. J., Thomson J., Colley S., Meysman F. J. R. and Slomp C. P. (2002). Oxidation and origin of organic matter in surficial Eastern Mediterranean hemipelagic sediments. Aquat. Geochemistry 8, 153–175. doi: 10.1023/A:1024271706896

Wanninkhof, R. (2014). Relationship between Wind Speed and Gas Exchange over the Ocean Revisited. Limnol. Oceanogr. Methods. 12 (JUN), 351–362. doi: 10.4319/lom.2014.12.351.

Whiticar, M. J. (1999). Carbon and hydrogen isotope systematics of bacterial formation and oxidation of methane. Chem. Geol. 161, 291–314. doi: 10.1016/S0009-2541(99)00092-3.

Wiesenburg, D. A.; Guinasso, N. L. (1979). Equilibrium Solubilities of Methane, Carbon Monoxide, and Hydrogen in Water and Sea Water. J. Chem. Eng. Data. 24 (4), 356–360. doi: 10.1021/je60083a006.
